# A comparison of tumour characteristics of screen-detected and interval cancers and their relationship with breast density and AJCC prognostic stage in a UK screening cohort

**DOI:** 10.1007/s00330-026-12593-6

**Published:** 2026-05-06

**Authors:** Fleur Kilburn-Toppin, Joshua Rothwell, Nicholas R. Payne, Yuan Huang, Sarah E. Hickman, Fiona J. Gilbert

**Affiliations:** 1 Department of Radiology, University of Cambridge School of Clinical Medicine, Cambridge, England; 2 Department of Radiology, Cambridge University Hospital NHS Foundation Trust, Cambridge, England; 3 EPSRC Cambridge Mathematics of Information in Healthcare Hub, University of Cambridge, Cambridge, England; 4 Department of Radiology, Barts Health NHS Trust, The Royal London Hospital, London, England

**Keywords:** Breast cancer, Mammography, Screening, Interval cancers, breast cancer prognosis

## Abstract

**Objectives:**

To compare tumour characteristics of screen-detected and interval cancers and their relationship with breast density and AJCC prognostic stage.

**Materials and methods:**

In this retrospective study, women screened with mammography between 1/4/2017 and 30/3/2020 at one site were included. Tumour characteristics were compared for screen-detected and interval cancers with breast density (Volpara) and AJCC (8th edition) prognostic stage for interval cancers. Categorical variables were compared using Pearson’s χ², Fisher’s exact, or binomial tests.

**Results:**

From 55,010 attendees, 723 cancers were diagnosed (463 screen-detected; 260 interval). Time to interval cancer diagnosis was longer in women with dense breasts compared with non-dense breasts (median: 767 vs 624 days; *p* = 0.04). Earlier stage interval cancers ( < IIA) were more likely diagnosed in the second and third year compared to year 1 ((33/78) 48.7% and (56/129) 43.4% vs (12/53) 22.6%; *p* = 0.02). Retrospectively missed interval cancers were less frequent in women with dense breasts compared with true interval cancers (41.4% vs 71.9%); *p* < 0.01. Retrospectively missed intervals were more likely than true intervals to be grade 3 ((15/29) 51.7% vs (64/196) 32.7%, *p* = 1.00), lymph node positive ((12/29) 41.4% vs (70/196) 35.7%, *p* ≥ 0.7) and ≥ stage IIA ((14/29) 48.3% vs (72/196) 36.7%, ≥ IIA, *p* = 0.68).

**Conclusions:**

The time to interval cancer diagnosis was longer for women with dense breasts. Interval cancers presenting within the first year after screening had the worst prognosis. Retrospectively missed interval cancers had worse prognostic features and were more likely in non-dense breasts.

**Key Points:**

***Question*** *To establish the relationship between screen-detected and interval cancers and breast density, and the prognosis of interval cancers depending on time of diagnosis and radiological signs.*

***Findings*** *Interval cancers presented later in women with dense breasts, but prognostically worse cancers presented earlier and were more likely to demonstrate retrospectively visible mammographic signs.*

***Clinical relevance*** *Our finding that prognostically worse interval cancers were most likely in the first year after screening, and when radiological signs were retrospectively present, identifies an important opportunity for AI-based tools to enhance screening programmes.*

**Graphical Abstract:**

**Figure:**
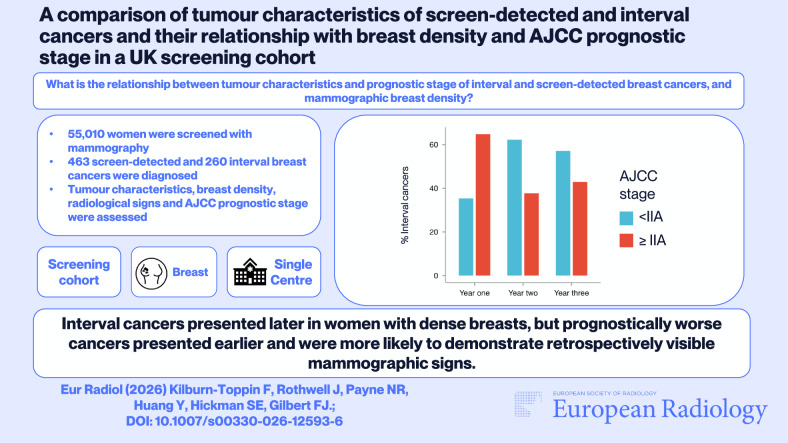

## Introduction

Breast cancer is the leading cause of global cancer incidence, with an estimated 2.3 million new diagnoses in 2020 [1]. National screening programmes, such as the UK triennial programme, aim to reduce breast cancer morbidity and mortality of breast cancer through early detection [2]. Mammographic breast density is a major risk factor; women with the highest density have a fourfold relative risk of breast cancer compared with those with the lowest [3].

High density is also a risk factor for breast ‘interval’ cancers—defined as those diagnosed between regular screening examinations [4]. Several studies have found that mammographic sensitivity reduces with increasing density [5, 6]. Density can be assessed visually using classifications such as the American College of Radiologists (ACR) Breast Imaging Reporting and Data System (BI-RADS) Atlas Fifth Edition four-point scale [7], or measured by automated methods [8]. The higher incidence of interval cancers in women with denser breasts is attributed to two factors: a ‘masking’ effect, when dense tissue obscures tumours [5, 9], and a biological effect related to tumour growth rate [10]. These cancers are particularly concerning because they are typically more aggressive than screen-detected cancers. Previous studies have found that interval cancers are larger than screen-detected cancers, with higher histologic grade and TNM stage, and more are frequently have a triple-negative phenotype [11–13]. Although the clinicopathological differences between interval and screen-detected cancers are well established, their association with density is less clear and remains inconsistent in the literature, with some studies finding interval cancers in low-density breasts to have a particularly aggressive phenotype and a worse prognosis [14–16]. This has important implications for the design of breast screening programmes based on density, as advocated by the European Society of Breast Imaging [17].

On retrospective review, interval cancers can be classified as either true positive (not present on the prior screening mammogram) or retrospectively missed (cancer visible in retrospect and missed at screening). Retrospectively missed cancers may account for up to 30% of cases [18, 19]. Multiple factors may contribute to interval cancers, including high density, which can mask cancers on mammography [20]. This has implications for the development of artificial intelligence (AI) techniques that could help radiologists identify mammographic findings that may be otherwise overlooked or dismissed as benign.

Advanced breast cancer is recognised as an important endpoint for determining breast cancer screening effectiveness. The American Joint Committee on Cancer (AJCC) latest eighth edition prognostic pathologic staging system includes anatomic staging elements plus tumour grade and oestrogen receptor (ER), progesterone receptor (PR), and human epidermal growth factor receptor 2 (HER2) status. In order to identify women at increased risk of breast cancer death who may benefit from earlier detection of aggressive tumours, having a definition of advanced cancer that maximises sensitivity for identifying breast cancer death, whilst maximising specificity, is imperative [21].

Kerlikowske et al examined staging systems and thresholds for defining advanced breast cancer and found AJCC prognostic pathologic stage was more accurate than the anatomic stage for predicting breast cancer death [22]. Additionally, they found AJCC prognostic pathologic stage IIA or higher as the staging system and threshold that has the best balance of sensitivity and specificity for predicting breast cancer death and thus the best choice when evaluating breast cancer screening effectiveness. For this reason, we chose stage IIA as our threshold for defining worse-prognosis cancer for comparison.

The objectives of this study were to compare tumour characteristics of screen-detected and interval cancers, their relationship with breast density, and AJCC prognostic criteria of interval cancers in relation to time to diagnosis and retrospective radiological classification.

## Methods

All women screened at one UK site between April 1st 2017, and March 30th 2020, were retrospectively identified via the National Breast Screening System (NBSS) database and included in this study. In the UK, women aged 50–70 years are offered screening every 36 months, with mammograms read by two expert human readers. Interval cancers were defined as breast cancers diagnosed after a negative screening mammogram but before the next scheduled screening round, or within 40 months of screening. A 40-month threshold is used in the UK as screening services may not invite all women exactly within 36 months due to capacity issues or scheduling delays, and a 40-month threshold ensures that cancers occurring just after the 3-year mark are still captured. Screen-detected cancers were defined as breast cancers diagnosed after screening recall. Both interval and screen-detected cancers were identified through NBSS. NBSS does not record the method of detection of interval cancers, in almost all cases, this is due to symptomatic presentation. No supplemental imaging was used in this study period. Uptake rate for screening was on average 79% during this period. Race and ethnicity data were not available.

Tumour characteristics (type, grade, size, and lymph node status) were retrieved from both NBSS and local databases. Pathological assessment was performed according to routine clinical practice at Cambridge University Hospitals NHS Foundation Trust, UK. Tumour size was defined as the largest invasive or in situ focus; for women undergoing neoadjuvant treatment, the largest tumour diameter on MRI was used. In cases of bilateral interval cancers, the tumour with the most aggressive characteristics was included. Micro- or macrometastases in axillary lymph nodes were considered positive. Cancer molecular subgroups were defined according to the 2013 St. Gallen International Expert Consensus. The AJCC 8th edition prognostic pathological staging system was applied, with stage IIA used as the threshold for defining worse prognosis cancer [23].

Breast density was assessed using anonymised mammograms from the Cambridge Cohort–Mammography East-Anglia Digital Imaging Archive (CC-MEDIA) dataset, linked to the included study participants. CC-MEDIA includes screening data for the Cambridge and Huntingdon, Norfolk and Norwich NHS Breast Screening programmes, with ethical approval (Health Research Authority Research Ethics Committee: 20/LO/0104, HRA Confidentiality Advisory Group: 20/CAG/0009, and Public Health England Research Advisory Committee: BSPRAC_090). Radiographic breast density was automatically assessed using Volpara (v.3.2.0; Volpara Health Technologies Ltd). This software requires raw, uncompressed mammography data with DICOM headers containing exposure parameters, which are used by its physics-based model to compute percentage volumetric density (VBD) for each image. For each case, the maximum VBD at the breast level is converted into a volpara density grade (VDG), calibrated to correspond with BI-RADS 5th Edition breast densities: a, almost entirely fat (VDG < 3.5%); b, scattered fibroglandular densities (3.5% ≤ VDG < 7.5%); c, heterogeneously dense (7.5% ≤ VDG < 15.5%); and d, extremely dense (15.5% ≥ VDG). This model has been validated in several previous studies [24, 25]. ‘Non-dense’ breasts were defined as those assessed as categories a or b, and ‘dense’ breasts as categories c or d. Where raw mammographic data were not available, density was assessed from *For Presentation* data using Lunit Insight v1.1.7 (Lunit Inc.) with categorisation thresholds found using cases where both Volpara and Lunit scores were available.

Interval cancers were classified according to UK national screening criteria: Category 1 (satisfactory— normal or benign mammographic features), Category 2 (Satisfactory with learning points—seen with hindsight but difficult to perceive, and not obviously malignant), or Category 3 (Unsatisfactory—appearance clearly malignant). These categories were derived from NBSS data, for which an interval cancer review had been conducted in accordance with UK NHS Breast Screening Programme interval cancer review guidance (https://www.gov.uk/government/publications/breast-screening-interval-cancers).

Categorical variables, such as the proportion of screen-detected and interval cancers in dense breasts, were compared using Pearson’s χ² test, Fisher’s exact test when expected counts were ≤ 5, or a binomial test when comparing two subgroup proportions. Continuous variables (time-to-diagnosis and tumour diameters) were assessed for normality using quantile-quantile plots and were compared between groups using Mann–Whitney *U*-tests. Analyses were conducted by J.R. and Y.H. using R (version 4.4.3; R Foundation for Statistical Computing). A two-sided *p*-value < 0.05 was considered statistically significant. Where appropriate, *p*-values were adjusted using the Holm method. For subgroup comparisons with limited sample sizes, we report unadjusted *p*-values to prioritise sensitivity over specificity, given insufficient power to detect clinically meaningful differences in these rare outcomes.

## Results

55,010 women underwent screening mammography between April 1st 2017, and March 31st 2020 (year 1: 19,035, year 2: 20,831, year 3: 15,144). Median age of the women screened was 59 years (IQR: 54–65). For interval cancers, the median age was 54 years (IQR: 59–66); for screen-detected cancers, 62 years (IQR: 56–68). A total of 723 cancers were diagnosed, comprising 463 screen-detected and 260 interval cancers. Of the screen-detected cancers, the number detected each year was: year 1: 150, year 2: 192, year 3: 121, giving an overall cancer detection rate of 8.4/1000. The distribution of cancers by breast density was: a, 10.1% (*n* = 78); b, 39.8% (*n* = 288); c, 32.5% (*n* = 235); and d, 16.7% (*n* = 121). A significantly higher proportion of interval cancers than screen-detected cancers was observed in density categories c (42.1% vs 27.2%) and d (26.6% vs 11.2%) (Table 1).

**Table 1:** Comparison of screen-detected cancer and interval cancer proportions for each density category

Density	Screen-detected cancers	Interval cancers	*p*^a^
a	13.8% (64/463)	5.4% (14/259)	< 0.001
b	47.7% (221/463)	25.9% (67/259)	< 0.001
c	27.2% (126/463)	42.1% (109/259)	< 0.001
d	11.2% (52/463)	26.6% (69/259)	< 0.001

Percentages are accompanied by their respective fraction in parentheses. Density was unavailable for one of the 260 interval cancers and was therefore not included in this table

^a^ Pearson’s χ² test

Time to interval cancer diagnosis was significantly longer in women with dense breasts (median: 767 vs 624 days; *p* = 0.04). Almost half (49.4%) of all interval cancers presented after 24 months, with a majority of these occurring in women with dense breasts (72.7% vs 27.3%; *p* < 0.01) (Table 2).

**Table 2:** Comparison of median time to interval cancer diagnosis and year of presentation between breast density groups

	Median time-to-diagnosis (days)	Year one	Year two	Year three	
All densities	728 [417, 921]	20.5% (53/259)	31.1% (78/259)	49.4% (128/259)	
Individual density categories					
a	629 [378, 888]	5.7% (3/53)	6.4% (5/78)	4.7% (6/128)	
b	642 [298, 906]	41.5% (22/53)	20.5% (16/78)	22.7% (29/128)	
c	778 [527, 948]	28.3% (15/53)	46.2% (36/78)	45.3% (58/128)	
d	739 [423, 917]	24.5% (13/53)	26.9% (21/78)	27.3% (35/128)	
Grouped density categories					
Non-dense (a + b)	642 [319, 905]	47.2% (25/53)	26.9% (21/78)	27.3% (35/128)	
Dense (c + d)	767 [502, 934]	52.8% (28/53)	73.1% (57/78)	72.7% (93/128)	
AJCC stage					
< IIA		22.6% (12/53)	48.7% (38/78)	43.4% (56/129)	*p* = 0.022^a^
≥ IIA		41.5% (22/53)	29.5% (23/78)	32.6% (42/129)	*p* = 0.34
Not available		35.8% (19/53)	21.8% (17/78)	24.0% (31/129)	*p* = 0.16

Median time-to-diagnosis is accompanied by its interquartile range. Percentages are accompanied by their respective fraction in parentheses. Mammographic density was unavailable for one of the 260 interval cancers and was therefore not included in this table

^a^ Time-to-diagnosis was longer in dense breasts (767 vs 624 days; *p* = 0.04, Mann–Whitney *U*-test)

Compared with screen-detected cancers, interval cancers were larger (mean diameter: 21.5 mm vs 14 mm; *p* < 0.001), more frequently invasive (87.7% vs 76.7%; *p* < 0.001), and represented a higher proportion of grade 3 tumours (31.2% vs 15.3%; *p* < 0.001). DCIS accounted for 22.2% of screen-detected cancers and 11.5% of interval cancers (*p* < 0.01). Comparison of molecular subtypes showed that Luminal A cancers comprised the majority in both groups, with interval cancers demonstrating a higher proportion of HER2 positive (5.7% vs 3.4%; *p* = 0.25) and triple-negative (11.4% vs 5.6%; *p* = 0.02) cancers (Table 3).

**Table 3:** Comparison of screen-detected and interval cancer tumour characteristics

Characteristic	Screen-detected cancers	Interval cancers	*p*^a^
Median tumour diameter (mm)	14 [9, 20]	21.5 [14, 39]	< 0.001
Histological grade			
1	14.9% (69/463)	13.5% (35/260)	1.00
2	45.8% (212/463)	42.7% (111/260)	1.00
3	15.3% (71/463)	31.2% (81/260)	< 0.001
DCIS	22.2% (103/463)	11.5% (30/260)	< 0.01
Not available	1.7% (8/463)	1.2% (3/260)	1.00
Lymph node status			
Positive	16.3% (58/355)	36.4% (83/228)	< 0.001
Negative	78.9% (280/355)	52.2% (119/228)	< 0.001
Not available	4.8% (17/355)	11.4% (26/228)	< 0.01
Tumour subtype			
Luminal A	66.2% (235/355)	59.2% (135/228)	0.10
Luminal B (HER2−)	14.4% (51/355)	8.3% (19/228)	0.04
Luminal B (HER2+)	8.5% (30/355)	8.8% (20/228)	1.00
HER2+	3.4% (12/355)	5.7% (13/228)	0.25
Triple negative	5.6% (20/355)	11.4% (26/228)	0.02
Not available	2.0% (7/355)	6.6% (15/228)	< 0.01

Median tumour diameter is accompanied by the interquartile range. Percentages are accompanied by their respective fraction in parentheses. Of 463 screen-detected and 260 interval cancers, 355 and 228 were invasive, respectively, and included within lymph node status and tumour subtype counts

^a^ Mann–Whitney *U*-test for tumour diameter; Pearson’s χ² test for all other characteristics

When comparing interval cancer by year of diagnosis, cancers diagnosed in the first year were more likely to be grade 3, lymph node positive, HER2 positive, and triple-negative, although this did not reach statistical significance. Interval cancers diagnosed at earlier stages (< IIA) were more likely to present in the second (48.7%) or third (43.4%) year, compared with first (22.6%) following screening (*p* = 0.02). Although, the proportion of interval cancers presenting at later stages was higher in the first year after screening (41.5%) compared with the second (29.5%) and third years (32.6%), this difference was not found to be significant (*p* = 0.34) (Table 4).

**Table 4:** Comparison of interval cancer characteristics and time-to-diagnosis

Characteristic	Year one	Year two	Year three	*p*^a^
Total	20.4% (53/260)	30.0% (78/260)	49.6% (129/260)	< 0.001
Median tumour diameter (mm)	26 [18, 45]	19 [14, 39]	21 [14, 37]	0.16
Histological grade				
1	9.4% (5/53)	14.1% (11/78)	14.7% (19/129)	1.00
2	32.1% (17/53)	43.6% (34/78)	46.5% (60/129)	0.79
3	35.8% (19/53)	29.5% (23/78)	30.2% (39/129)	1.00
DCIS	20.8% (11/53)	11.5% (9/78)	7.8% (10/129)	0.04
Not available	1.9% (1/53)	1.3% (1/78)	0.8% (1/129)	1.00
Lymph node status				
Positive	43.9% (18/41)	36.7% (25/68)	33.6% (40/119)	1.00
Negative	43.9% (18/41)	55.9% (38/68)	52.9% (63/119)	1.00
Not available	12.2% (5/41)	7.4% (5/68)	13.4% (16/119)	1.00
Tumour subtype				
Luminal A	48.8% (20/41)	61.8% (42/68)	61.3% (73/129)	1.00
Luminal B (HER2−)	12.2% (5/41)	7.4% (5/68)	7.6% (9/129)	1.00
Luminal B (HER2+)	4.9% (2/41)	11.8% (8/68)	8.4% (10/129)	1.00
HER2+	7.3% (3/41)	4.4 (3/68)	5.9% (7/129)	1.00
Triple negative	22.0% (9/41)	7.4% (5/68)	10.1% (12/129)	0.38
Not available	4.9% (2/41)	7.4% (5/68)	6.7% (8/129)	1.00
AJCC stage				
< IIA	22.6% (12/53)	48.7% (38/78)	43.4% (56/129)	0.02^b^
≥ IIA	41.5% (22/53)	29.5% (23/78)	32.6% (42/129)	0.34
Not available	35.8% (19/53)	21.8% (17/78)	24.0% (31/129)	0.16

Median tumour diameter is accompanied by the interquartile range. Percentages are accompanied by their respective fraction in parentheses

^a^ Kruskal–Wallis tests for tumour diameters; Pearson’s χ² tests for all other characteristics

^b^ Years two and three were comparable (*p* = 0.48), and both were higher than year one (*p* ≤ 0.02)

Retrospective radiological classification of invasive interval cancers showed that 87% (*n* = 195) of cancers were classified as Category 1 (normal or benign mammographic features); 12% (*n* = 27) were classified as Category 2 (seen with hindsight, difficult to perceive, not obviously malignant), and one case was classified as Category 3 (obviously malignant). Of the 28 interval cancers classified as Category 2 or 3, i.e. ‘retrospectively missed’, 10 occurred in year 1, 8 in year 2 and 10 in year 3. Retrospectively missed cancers were more likely than Category 1 cancers (true positives) to be grade 3 (51.7% vs 32.7%), lymph node positive (41.4% vs 35.7%), and HER2 positive (10.3% vs 4.6%), and stage IIA or higher (48.4% vs 36.7%), although not reaching statistical significance. Retrospectively missed interval cancers also demonstrated a significant difference in density distribution compared with true positives, with fewer occurring in dense breasts (41.4% vs 71.9%; *p* < 0.01) (Table 5).

**Table 5:** Comparison of interval cancer characteristics and radiological classification

Characteristic	True positive interval cancers	Retrospectively missed interval cancers	*p*^a^
Total	87.1% (196/225)	12.9% (29/225)	< 0.001
Median tumour diameter (mm)	20 [14, 35]	32 [16, 42]	0.11
Histological grade			
1	15.8% (31/196)	13.8% (4/29)	1.00
2	51.0% (100/196)	34.5% (10/29)	0.14
3	32.7% (64/196)	51.7% (15/29)	0.07
Not available	0.5% (1/196)	0.0% (0/29)	1.00
Lymph node status			
Positive	35.7% (70/196)	41.4% (12/29)	1.00
Negative	53.1% (104/196)	48.3% (14/29)	1.00
Not available	11.2% (22/196)	10.3% (3/29)	1.00
Tumour subtype			
Luminal A	60.7% (119/196)	51.7% (15/29)	1.00
Luminal B (HER2–)	7.7% (15/196)	13.8% (4/29)	0.45
Luminal B (HER2+)	9.2% (18/196)	3.4% (1/29)	0.50
HER2+	4.6% (9/196)	10.3% (3/29)	0.40
Triple negative	11.7% (23/196)	10.3% (3/29)	1.00
Not available	6.1% (12/196)	10.3% (3/29)	0.65
AJCC stage			
< IIA	48.5% (95/196)	34.5% (10/29)	0.68
≥ IIA	36.7% (72/196)	48.3% (14/29)	0.68
Not available	14.8% (29/196)	17.2% (5/29)	0.78
Density			
a	3.6% (7/196)	20.7% (6/29)	< 0.01
b	24.0% (47/196)	37.9% (11/29)	0.17
c	43.9% (86/196)	20.7% (6/29)	0.03
d	28.1% (55/196)	20.7% (6/29)	0.54
Not available	0.5% (1/196)	0.0% (0/29)	1.00

Median tumour diameter is accompanied by the interquartile range. Percentages are accompanied by their respective fraction in parentheses. Of 228 invasive interval cancers, 3 were ‘Unclassifiable’ on retrospective assessment and not included in this table

^a^ Binomial test for total subgroup proportions, Mann–Whitney *U*-test for tumour diameter; Pearson’s χ² test for all other characteristics

When comparing screen-detected and interval cancers grouped by density (non-dense: a and b, vs dense: c and d), no significant differences were observed in tumour size, grade, lymph node positivity, molecular subtypes, or AJCC prognostic criteria between groups (Supplementary Material—Tables S1 and S2).

## Discussion

It is well established that high mammographic density reduces mammographic sensitivity, increasing the risk of interval cancers [5]. One-third of all cancers in our three-year screening cohort were interval cancers, with a higher proportion occurring in dense breasts and almost half of the interval cancers were diagnosed in year 3 after screening. This is consistent with the literature on triennial screening programmes, which report higher interval cancer rates than biennial programmes [26, 27]. In a review by Houssami et al, interval cancers represented around 17–30% of the cancers occurring in screening participants in biennial screening programmes [27]. That proportion was relatively lower for annual (14.7% based on one study) and higher for triennial (32–38%) screening intervals. They also demonstrated that interval cancer rates in year 2 are at least twice those in year 1. However, these findings should not be taken to infer that annual screening has better population outcomes than biennial screening, and the balance between the benefits and harms of different screening intervals is still a matter of debate. A systematic review performed by the European Commission Initiative on Breast Cancer addressed the question ‘Should an annual, biennial or triennial screening frequency be used for screening asymptomatic women?’ [28]. In women 50–69 years old, they concluded that annual screening compared to biennial screening may have small additional benefits but a significant increase in false positive results. Triennial compared to biennial screening suggests the latter provides more benefits but also some additional harms, namely increased interval cancers. Recommendations about mammography screening intervals may also depend on breast density, given higher interval cancer rates in this group. A study by Seely et al compared interval cancer rates in breast screening programs with a policy of annual vs those with biennial screening for women with dense breasts [29], demonstrating that annual compared with biennial screening in women with dense breasts had the greatest impact on reducing interval cancer rates.

The distribution of tumour characteristics between screen-detected and interval breast cancers was also consistent with the literature, with interval breast cancers being larger at diagnosis, of higher grade, and more likely to be lymph node positive and triple-negative [11, 13]. The interval cancer cohort showed a statistically higher proportion of triple-negative and HER2-positive cancers, which are associated with a worse prognosis, consistent with the literature [11–13].

In our study, we found a significantly longer time-to-diagnosis in women with dense breasts compared with those with non-dense breasts (Table 2). Previous studies have reported shorter tumour volume doubling times in very fatty breasts [30, 31]. However, there is a complex interplay between tumour doubling time and time to clinical presentation for interval cancers. Our findings contrast with those of Larsen et al, who reported that women with extremely dense breasts experienced shorter times from screening to diagnosis of interval cancer compared with women in other density categories (median 427 days for BI-RADS 4 vs 482–496 days for BI-RADS 1–3) [32]. They noted the rationale for this observation was unclear, but suggested it may relate to the masking effect at the time of imaging interpretation.

We found no significant differences in tumour characteristics or molecular subtype by density for either screen-detected or interval cancers, which is consistent with most published studies. Whether density gives rise to more highly proliferative tumours remains uncertain. While some studies have reported an association between higher density and more aggressive tumour characteristics [33–35], most have found no such association [36–38]. Eriksson et al found that women diagnosed with interval cancers in non-dense breasts ( < 25% density) appeared to have a particularly aggressive phenotype and worse prognosis compared with women with screen-detected cancers in dense breasts (> 75%). Similarly, Holm et al reported that interval cancers in women with low breast density were associated with a worse prognosis, suggesting that interval cancers in non-dense breasts are more likely to represent aggressive true intervals [14]. In another study including 2582 cancers (both screen-detected and interval cancers), Bare et al found that triple-negative interval cancers were more common than other phenotypes in breasts with low mammographic density (> 25%) [39]. Some of the discrepancies between studies may be attributable to differences in study design or different definitions of breast density. Importantly, several studies do not account for whether cancers were detected by screening, which is important to prevent overestimating the association between tumour phenotype and breast density.

In our study, more aggressive tumours with worse prognostic criteria were observed in the first year after screening compared with the second and third years (Table 4). Findings reported in the literature are again inconsistent. Both Eriksson et al and Holm et al reported no difference in interval cancers detected within one year of a negative screen vs those detected in the second year of a biennial screening programme [14, 15]. Larsen et al reported that invasive interval cancers diagnosed within the first year appeared subjectively less aggressive than those detected in the second year for women with category d density, with triple-negative cancers accounting for double the proportion of interval cancers found in the second year (19.7%) than the first (8.6%). However, the study lacked sufficient statistical power to determine if this difference was significant [29]. These differences may again be attributable to differing screening round lengths, as the majority involved biennial programmes.

In our cohort, true positive interval cancers accounted for 87.1% of cases, whereas retrospectively missed interval cancers comprised 12.9% (Table 5). This aligns with the UK screening programme reported rates, in which approximately 20% of interval cancers are classified as retrospectively missed cancers despite double reading [40]. Several recognised technological and human factors can contribute to breast cancers being missed on mammography, including poor image quality or suboptimal positioning, benign-appearing lesions, slowly developing asymmetries, as well as information overload and perceptual errors [20].

The absence of a standardised method for radiological classification of interval cancers, combined with the inherently subjective nature of mammography interpretation, hampers valid comparisons between screening programmes. Houssami et al, in their review of the literature, reported that the proportion of retrospectively missed interval breast cancers ranged from 13.6% to 35.0%, with most studies reporting a frequency of 20 to 25% based on radiological surveillance [27]. Furthermore, a study from Ciatto and colleagues demonstrated that increasing the information available to screen-readers significantly increased the proportion of interval breast cancers classified as missed, highlighting the impact of review methodology on radiological classification [41].

While some studies have reported no significant differences in histopathological characteristics between interval cancers classified as true interval cancers and retrospectively missed cancers [42], our finding that retrospectively missed interval cancers are larger than true interval cancers aligns with the majority of studies [27], likely due to a delay in detection. Consistent with our observation that retrospectively missed interval cancers had higher nodal positivity rates, Nykänen et al reported that positive nodal status was observed in up to 53.6% of missed interval cancers—the highest level of nodal spreading among all interval cancer subgroups [43]. Similarly, our finding of 71.9% of true positive interval cancers being in dense breasts, compared with 41.4% of retrospectively missed cancers (Table 5), is in agreement with the results reported by Domingo et al—who reported that occult tumours (true positives, equivalent to UK Category 1) had similar phenotypic characteristics to screen-detected cancers, with high breast density being strongly associated with occult tumours [16]. Minimal-sign cancers (equivalent to UK Category 2) were biologically similar to true interval cancers but showed no association with breast density. Consistent with our findings, Blanche et al demonstrated that mammographic density was associated with occult breast cancers rather than missed interval cases, highlighting a probable masking effect [44].

The significance of retrospectively missed cancers being worse prognostically is of importance, as results from AI cancer detection algorithms have shown promising results for earlier cancer detection, and thus a decrease in interval cancers [45, 46]. If AI algorithms are able to detect the more subtle signs that human readers miss, then it is even more beneficial if these are cancers that are likely to have a greater impact on mortality. Shortening the screening interval along with the use of AI has the potential to reduce interval cancer rates by higher cancer detection rates, and by identifying women who may benefit from more frequent imaging and/or supplemental imaging.

Our study has several strengths. We analysed a large, well-described cohort of screen-detected and interval cancers from the UK three-year screening programme, including molecular subtype information. We utilised automated density measurements, enabling a more objective assessment of the impact of breast density on prognostic factors for interval and screen-detected cancers than would be possible with subjective radiological classification. Furthermore, classification of interval cancers as true negatives or false positives was derived from NBSS classification, performed independently by two radiologists as part of the mandatory interval cancer review conducted in each unit, thereby minimising bias in this study.

Our study also had limitations. First, its retrospective nature introduces potential selection biases inherent to non-prospective work. Second, raw data were not available for all mammograms, necessitating the use of two different density tools rather than only Volpara. However, density scores were adjusted to match the Volpara density distribution previously reported by Payne et al [5]. Thirdly, the majority of studies available for comparison related to biennial rather than triennial screening programmes. Finally, due to the limited number of interval cancers available for molecular subtype analysis, statistical significance could not be determined.

## Conclusion

Whilst we found no association between breast density, tumour aggressiveness or AJCC stage, the majority of interval cancers diagnosed in years 2 and 3 after screening were in women with dense breast tissue. Almost half of interval cancers presented in year 3 after screening, which is a recognised disadvantage of triennial screening programmes, and more frequent screening will reduce interval cancer rates. However, we found that worse prognosis cancers, defined as stage IIA and above, were more likely to present in the first year after screening and when radiological signs are present. This highlights an important opportunity for AI-based tools and supplemental imaging to enhance screening programmes and ultimately improve breast cancer outcomes.

## Supplementary information

ELECTRONIC SUPPLEMENTARY MATERIAL

## Abbreviations

BI-RADS: Breast Imaging Reporting and Data System
AI: Artificial intelligence
AJCC: American Joint Committee on Cancer
CC-MEDIA: Cambridge Cohort–Mammography East-Anglia Digital Imaging Archive
HER2: Human epidermal growth factor receptor 2
NBSS: National Breast Screening System
VDG: Volpara density grade
