# Supplementary material for: A comparison of tumour characteristics of screen-detected and interval cancers and their relationship with breast density and AJCC prognostic stage in a UK screening cohort
Source: Eur Radiol. 2026 May 6;36(9):7221–30. doi: 10.1007/s00330-026-12593-6 (PMC13451422; doi:10.1007/s00330-026-12593-6)
Supplement: Supplementary file 1 [file 330_2026_12593_MOESM1_ESM.pdf]

A comparison of tumour characteristics of screen-detected and interval cancers and their relationship with breast density and AJCC prognostic stage in a UK Screening Cohort

ELECTRONIC SUPPLEMENTARY MATERIAL

Characteristic	Non-dense (a+b)	Dense (c+d)	*p* †
Total	61.6% (285/463)	38.4% (178/463)	**< 0.001**
Median tumour diameter (mm)	13.5 [9, 20]	15 [10, 23]	0.17
Histological grade			
1	14.7% (42/285)	15.2% (27/178)	1.00
2	48.1% (137/285)	42.1% (75/178)	1.00
3	16.8% (48/285)	12.9% (23/178)	1.00
DCIS	18.6% (53/285)	28.1% (50/178)	**9.02**
Not available	1.8% (5/285)	1.7% (3/178)	1.00
Lymph node status			
Positive	16.6% (38/229)	15.9% (20/126)	1.00
Negative	78.6% (180/229)	79.4% (100/126)	1.00
Not available	4.8% (11/229)	4.8% (6/126)	1.00
Tumour subtype			
Luminal A	67.2% (154/229)	64.3% (81/126)	1.00
Luminal B (HER2–)	12.2% (28/229)	18.3% (23/126)	0.92
Luminal B (HER2+)	9.2% (21/229)	7.1% (9/126)	1.00
HER2+	3.9% (9/229)	2.4% (3/126)	1.00
Triple negative	5.2% (12/229)	6.3% (8/126)	1.00
Not available	2.2% (5/229)	1.6% (2/126)	1.00

Commented [YH1]: For this comparison you don't have a 2x2 table and cannot use a chi square test, which examines the proportion of one variable conditioned to another variable. Here you should be using a binomial test instead. binom.test(c(285, 178), p=0.5) in R returns p<0.0001. Make sure you describe this in the methods section.

Table S1: Comparison of screen-detected cancer tumour characteristics in different breast densities

Median tumour diameter is accompanied by interquartile range. Percentages are accompanied by their respective fraction in parentheses. Of 463 screen-detected cancers, 355 were invasive and included within lymph node status and tumour subtype counts.

† Binomial test for total subgroup proportions, Mann-Whitney U test for tumour diameter; Pearson's χ^2 test for all other characteristics.

Characteristic	Non-dense (a+b)	Dense (c+d)	*p* †
**Total**	31.3% (81/259)	68.7% (178/259)	**< 0.001**
**Median tumour diameter(mm)**	22 [14, 40]	21 [15, 38]	0.75
**Histological grade**			
**1**	17.3% (14/81)	11.8% (21/178)	1.00
**2**	35.8% (29/81)	46.1% (82/178)	0.79
**3**	35.8% (29/81)	28.7% (51/178)	1.00
**DCIS**	11.1% (9/81)	11.8% (21/178)	1.00
**Not available**	0.0% (0/81)	1.7% (3/178)	1.00
**Lymph node status**			
**Positive**	37.5% (27/72)	36.1% (56/155)	1.00
**Negative**	50.0% (36/72)	53.5% (83/155)	1.00
**Not available**	12.5% (9/72)	10.3% (16/155)	1.00
**Tumour subtype**			
**Luminal A**	61.1% (44/72)	58.7% (91/155)	1.00
**Luminal B (HER2–)**	9.7% (7/72)	7.7% (12/155)	1.00
**Luminal B (HER2+)**	6.9% (5/72)	9.0% (14/155)	1.00
**HER2+**	4.2% (3/72)	6.5% (10/155)	1.00
**Triple negative**	12.5% (9/72)	11.0% (17/155)	1.00
**Not available**	5.6% (4/72)	7.1% (11/155)	1.00

Table S2: Comparison of invasive interval cancer tumour characteristics in different breast densities

Median tumour diameter is accompanied by interquartile range. Percentages are accompanied by their respective fraction in parentheses. Mammographic density was unavailable for one of the 260 interval cancers and was therefore not included this table; of these, 227 were invasive and included within lymph node status and tumour subtype counts.

† Binomial test for total subgroup proportions, Mann-Whitney U test for tumour diameter; Pearson's χ^2 test for all other characteristics.
